# Application of the Speed-Duration Relationship to Normalize the Intensity of High-Intensity Interval Training

**DOI:** 10.1371/journal.pone.0076420

**Published:** 2013-11-14

**Authors:** Carrie Ferguson, John Wilson, Karen M. Birch, Ole J. Kemi

**Affiliations:** 1 School of Biomedical Sciences, Faculty of Biological Sciences, University of Leeds, Leeds, United Kingdom; 2 Institute of Cardiovascular and Medical Sciences, College of Medical, Veterinary and Life Sciences, University of Glasgow, Glasgow, United Kingdom; The University of Queensland, Australia

## Abstract

The tolerable duration of continuous high-intensity exercise is determined by the hyperbolic Speed-tolerable duration (S-t_LIM_) relationship. However, application of the S-t_LIM_ relationship to normalize the intensity of High-Intensity Interval Training (HIIT) has yet to be considered, with this the aim of present study. Subjects completed a ramp-incremental test, and series of 4 constant-speed tests to determine the S-t_LIM_ relationship. A sub-group of subjects (n = 8) then repeated 4 min bouts of exercise at the speeds predicted to induce intolerance at 4 min (WR_4_), 6 min (WR_6_) and 8 min (WR_8_), interspersed with bouts of 4 min recovery, to the point of exercise intolerance (fixed WR HIIT) on different days, with the aim of establishing the work rate that could be sustained for 960 s (i.e. 4×4 min). A sub-group of subjects (n = 6) also completed 4 bouts of exercise interspersed with 4 min recovery, with each bout continued to the point of exercise intolerance (maximal HIIT) to determine the appropriate protocol for maximizing the amount of high-intensity work that can be completed during 4×4 min HIIT. For fixed WR HIIT t_LIM_ of HIIT sessions was 399±81 s for WR_4_, 892±181 s for WR_6_ and 1517±346 s for WR_8_, with total exercise durations all significantly different from each other (P<0.050). For maximal HIIT, there was no difference in t_LIM_ of each of the 4 bouts (Bout 1: 229±27 s; Bout 2: 262±37 s; Bout 3: 235±49 s; Bout 4: 235±53 s; P>0.050). However, there was significantly less high-intensity work completed during bouts 2 (153.5±40. 9 m), 3 (136.9±38.9 m), and 4 (136.7±39.3 m), compared with bout 1 (264.9±58.7 m; P>0.050). These data establish that WR_6_ provides the appropriate work rate to normalize the intensity of HIIT between subjects. Maximal HIIT provides a protocol which allows the relative contribution of the work rate profile to physiological adaptations to be considered during alternative intensity-matched HIIT protocols.

## Introduction

In classic epidemiological data it is well established that there are significant health benefits associated with leading a physically active lifestyle (e.g. [1], [2], [3]). This assertion is further strengthened by the demonstration that training interventions can increase the maximal rate of pulmonary oxygen uptake (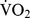
_max_) (a primary measure of physical fitness/exercise capacity and performance, and a strong predictor of all-cause mortality [4], [5]) (e.g. [6], [7]); and improve both metabolic and cardiovascular function when integrated as part of a lifestyle intervention or rehabilitation program (e.g. [8], [9], [10], [11]). Hence, exercise training has the capacity to improve both performance/exercise tolerance and reduce risk factors for both metabolic and cardiovascular disease. Therefore, given the implications of training for improving exercise performance, and in the prevention/rehabilitation of chronic disease, establishing optimal training strategies – not only to maximize training adaptations and associated health-related benefits, but also to improve participation and adherence in the general population – is of critical importance.

Key in this regard is the intensity of the exercise. It has been suggested that improvements in physiological functioning resulting from exercise training exist on a continuum [12], [13], [14], such that continuous higher-intensity exercise leads to greater benefits than that of a moderate-intensity [6], [15], [16], [17]. However, accumulation of high volumes of continuous, progressively higher intensity exercise is limited by the mechanisms that result in rapid exercise intolerance – i.e. tolerable duration is intensity dependent [18], [19]. This has led to significant interest in High-Intensity Interval Training (HIIT). Repeated short-duration (i.e. ∼30 s) all-out Wingate-style HIIT; i.e. Sprint Interval Training (SIT) is popular, and has been demonstrated to effectively improve endurance capacity and time-trial performance [20], [21], [22], [23], muscle oxidative enzyme activity [20], [21], [22], [23] and aerobic capacity (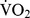
_max_) ([21], [24]), as well as specific health-related parameters such as insulin sensitivity [24], [25], blood pressure [24] and vascular function [26] in a time-efficient manner (compared with current moderate-intensity physical activity guidelines; i.e. 150 min/week; [27]).

Despite the significant evidence demonstrating benefits in both health and performance related parameters with short-duration (i.e. ∼30 s) SIT, there is evidence to suggest there may be similar, or even greater benefits attained from lowering the absolute work rate, prolonging the duration of the high-intensity interval (i.e. ∼4 min) and performing this as either an all-out sprint (matched for total work with a SIT session; [28]), or at a constant WR, in both health and disease (e.g. [10], [11], [12], [29], [30]). However, when high-intensity constant-load exercise bouts are extended beyond ∼2 min, exercise tolerance is shaped by the hyperbolic Power-tolerable duration (P-t_LIM_) relationship (analogous to the Speed-tolerable duration (S-t_LIM_) relationship in treadmill exercise) [18], [31]. The P-t_LIM_ relationship is therefore of critical significance when trying to identify the correct WR for an HIIT protocol in which the exercise bouts are prolonged.

In the P-t_LIM_ model, once a critical threshold (i.e. the critical power (CP) or critical speed (CS)) is exceeded – with this the asymptote of the P-t_LIM_ relationship which represents the upper limit for which a steady-state in 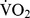, arterial blood acid-base status and intramuscular phosphocreatine and inorganic phosphate can be attained [18], [32] – tolerable duration is predictably determined by the rate at which a fixed quantity of work above the CP asymptote is performed. This fixed quantity of supra-CP work is termed W′ (cycle ergometry) or D′ (treadmill exercise), with this hypothesized to reflect either a fixed energy store associated with O_2_ deficit-related mechanisms (i.e. muscle phosphocreatine, stored O_2_, glycolysis/glycogenolysis) or the accumulation of related fatigue metabolites (e.g. intramuscular inorganic phosphate and H^+^, interstitial K^+^) to a fixed critical limit [18], [32], [33]. As the asymptote (CP) of the hyperbolic P-t_LIM_ relationship does not change with prior exercise [34], [35], subsequent high-intensity (supra-CP) exercise tolerance is therefore determined by the balance between the extent of W′ depletion in the preceding bout and subsequent W′ repletion during the intervening recovery period [34], [35], [36].

Despite this, there has been little consideration of the P-t_LIM_ relationship when determining the ‘intensity’ (or more correctly, the work rate) for HIIT that is comprised of exercise bouts longer than ∼2 min, with studies typically defining the work rate used based on % HR_max_ (∼95% HR_max_; [10], [11], [12], [30]) or % 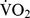
_max_ (∼90% 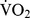
_max_; [29], [37], [38]). However, as CP does not occur at a fixed % of HR_max_ or 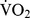
_max_ [19] and W′ does not represent the same volume of supra-CP exercise in all individuals (e.g. [39]) these approaches are sub-optimal. The consequence is that the metabolic stress and thus the exercise intensity experienced during the HIIT program will be variable between participants unless the P-t_LIM_ is accounted for. However, given the proposed relationship between intensity and both health- and performance-related fitness benefits [12], [13], [14], and the potential for the duration of the high-intensity exercise bout to have an impact on the training adaptations [28], the P-t_LIM_ relationship should be taken into account when normalizing the intensity of HIIT to appropriately investigate these assertions.

HIIT protocols comprising 4×4 min bouts are commonly used in both health and disease as a viable, more effective training protocol than traditional moderate-intensity interventions (e.g. [10], [11], [12], [29], [30]). Hence, the purpose of this investigation was to determine the appropriate constant-WR for a 4×4 min HIIT that would allow for the completion of the desired 4×4 min bouts, normalizing the intensity of HIIT between individuals, and then consider how the P-t_LIM_ relationship can be applied to maximize the volume of high-intensity work that can be completed in 4×4 min bouts in a HIIT program, thus making longer duration HIIT analogous to SIT (i.e. all-out effort in each bout) and providing a method to consider the relative importance of the work rate profile during intensity-matched training to the physiological adaptations. We hypothesized that for constant-WR HIIT the P-t_LIM_ relationship can be used to identify the WR that normalizes the intensity of a 4×4 min HIIT protocol. In addition, we hypothesized that W′ depletion and subsequent W′ repletion occurs at a fixed rate, allowing the P-t_LIM_ relationship to be used to maximize the volume of work that can be completed in a 4×4 min HIIT program.

## Methods

### Subjects

A total of 11 healthy, recreationally active males (mean ± standard deviation (SD); age 23±4 yr; height 178±5 cm; mass 72±5 kg) who met the inclusion criteria (i.e. recreationally active males, aged 18–35 yr who were free from illness or any medical condition) volunteered, and provided written informed consent to participate in the study (as approved by the Faculty of Biomedical and Life Sciences Ethical Committee for non-clinical research, University of Glasgow, in accordance with the Declaration of Helsinki). All subjects were well accustomed to high-intensity exercise. Although none of the subjects were participating in competitive training at the time of the study 2 subjects had a running background, with the others involved in recreational running training. Following familiarisation with all equipment, protocols and procedures, subjects visited the laboratory on at least 6 separate occasions, each at a similar time of day, with at least 24 hr between each test. Each individual participated in no more than 3 experimental sessions in any given week. For each test, subjects were instructed to arrive rested (no strenuous exercise in the previous 24 hr), and having abstained from alcohol (24 hr), food (2 hr minimum) and caffeine ingestion (4 hr) prior to each test. Throughout the study participants were asked to consume their normal diet, and prior to all testing, arrive at least 2 hr postprandial having consumed a normal, healthy meal.

### Equipment and measurements

All exercise tests were conducted on a motor driven programmable treadmill (PPS Med, Woodway, Weil am Rhein, Germany) set at a gradient of 1% to take into account the lack of air resistance with indoor treadmill running, and thus match the energetic cost of the treadmill exercise with that of outdoor running [40]. During all tests subjects breathed through a mouthpiece connected to a large 2-way non-rebreathing valve (2700 series, Hans Rudolph, Shawnee, KS, USA), allowing collection of the respired gas (via a 1.5 m length of 3.5 cm diameter tubing) in a Douglas bag. This allowed measurement of the expired gas concentrations (Paramagnetic (O_2_) and Infrared (CO_2_) analyzers; Servopro 4100 gas analyzer, Servomex, Crowborough, UK) and gas volume (Dry gas meter; Harvard Apparatus, Edenbridge, UK), thus allowing calculation of gas exchange variables (specifically 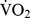). Prior to each test the gas analyzers were calibrated in accordance with manufacturers guidelines using precision analyzed gases which spanned the physiological range of inspired and expired gas concentrations, with gas mixtures re-sampled post-test to confirm stability in relation to the initial gas calibration.

Throughout all tests heart rate (HR) was measured and recorded every 5 s using a short-range telemetry HR monitor (S610i, Polar Electro Oy, Kempele, Finland). At specific time points in all protocols a small sample (approximately 25 µl) of capillary blood was obtained from the fingertip of the heated hand and analyzed immediately post-test for whole-blood [lactate] ([L^−^]) using an automated analyzer (GM7, Analox Instruments, London, UK). The analyzer was calibrated using an 8 mM standard L^−^ solution, the concentration of which was also checked post-test to confirm the validity of the measurements obtained.

### Exercise protocols

**Figure 1:**
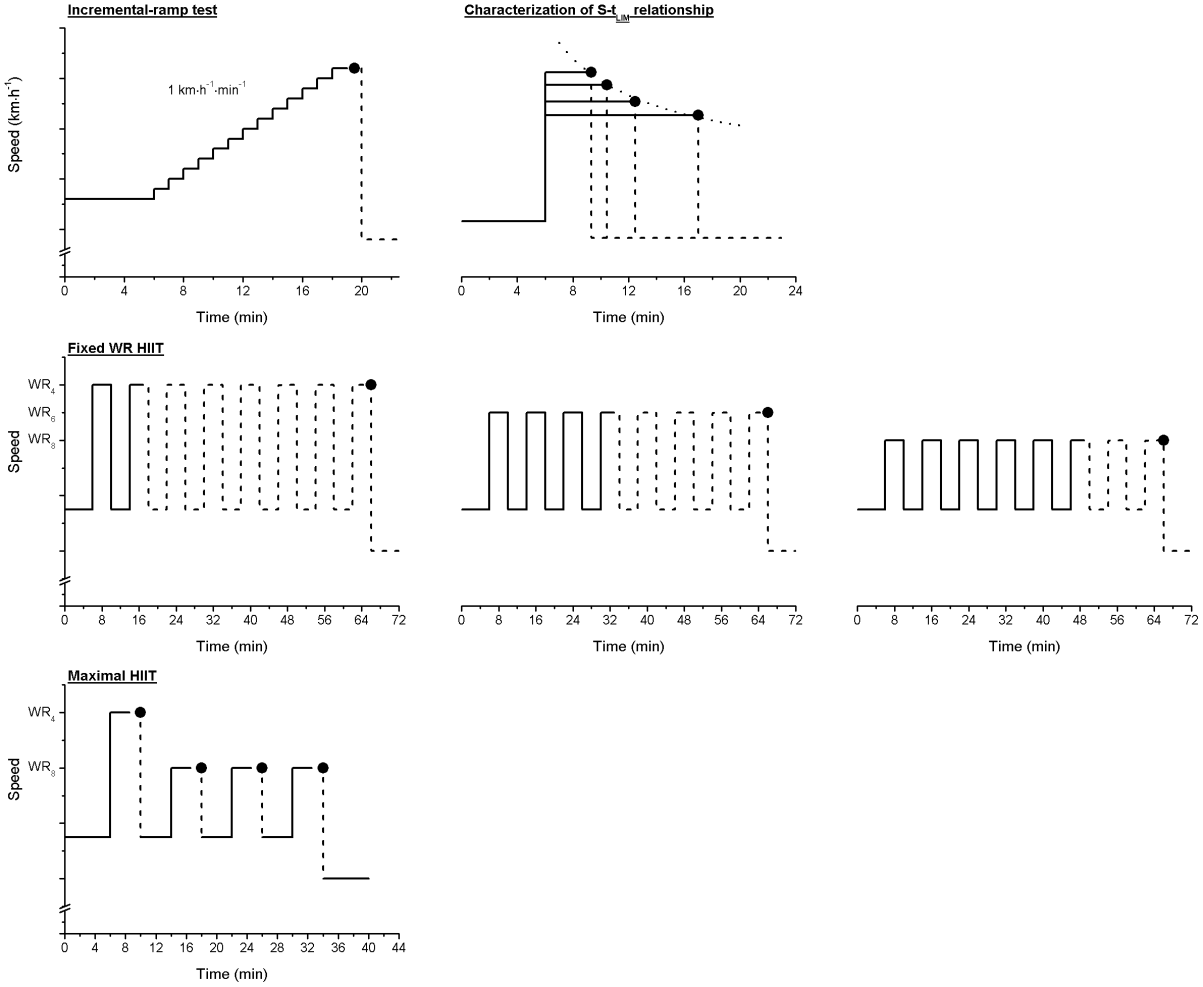
Schematic of the treadmill speed profiles performed during the Incremental-ramp test, the constant-speed tests for characterization of the Speed-tolerable duration (S-t_LIM_) relationship – dotted line (top left and right respectively), the fixed HIIT protocols in which 4 min bouts at WR_4_, WR_6_ or WR_8_ were alternated with 4 min recovery bouts until the limit of tolerance was attained (middle row), and the maximal HIIT protocol in which each bout was performed to the limit of tolerance, with a fixed 4 min recovery between each bout (bottom). • represents the limit of exercise tolerance in all protocols.

All exercise tests were preceded by a period of at least 6 min brisk walking at a speed of 5.5 km·h^−1^ (with the exception of the incremental-ramp test; see below for details), and concluded with a period of 6 min walking at a speed of 4.0 km·h^−1^. For each test, subjects were instructed to run as long as possible (i.e. to the point of exercise intolerance), and at the point at which they could not longer maintain the set treadmill speed – despite strong verbal encouragement – they were instructed to support their weight on the handrails and straddle the treadmill. At this point (i.e. exercise intolerance) the speed of the treadmill was immediately reduced, and the 6 min cool-down at 4.0 km·h^−1^ commenced. For a schematic of the exercise protocols please refer to Figure 1.

#### Incremental-ramp test

This test, to exercise intolerance, was performed to determine peak 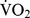 (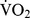
_peak_), and establish an appropriate starting speed to characterize the S-t_LIM_ relationship (see below). In the incremental-ramp test, following a period of 6 min running at 8 km·h^−1^, speed was increased at a rate of 1 km·h^−1^·min^−1^, until the point of exercise intolerance (Figure 1). Once the subject was considered to be close to the point of exercise intolerance, serial expired gas samples of a 60 s duration were collected in Douglas bags to ensure 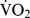
_peak_ was captured. In the event that the limit of tolerance was obtained less than ∼20 s into the gas collection, the value obtained from the previous 60 s gas collection was assumed to be 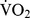
_peak_.

#### Characterization of the Speed-tolerable duration (S-tLIM) relationship

A randomized series of four separate constant-speed tests were conducted across a range of speeds selected to induce intolerance within a duration of ∼3–20 min [18]. During these tests the treadmill speed was rapidly increased to that required (treadmill acceleration 0.72 km·h^−1^·s^−1^, 0.200 m·s^−1^·s^−1^) from the 5.5 km·h^−1^ baseline, with subjects instructed to continue running at this speed until the point of exercise intolerance (Figure 1). From these tests the S-t_LIM_ relationship was characterized, with CS (intercept) and D′ (slope), the parameters of this relationship, estimated using least-squares linear regression of the linear S-t_LIM_^−1^ relationship (i.e. S = (D′/t_LIM_)+CS) [18]. Following estimation of the parameters of the S-t_LIM_ relationship within acceptable limits (defined as the standard error (SE) of the estimate being less than 2% for CS and 10% for D′; requiring additional tests at a different speed in 2 subjects), the speeds predicted to induce exercise intolerance at 4 min (WR_4_), 6 min (WR_6_) and 8 min (WR_8_) were derived by interpolation of the S-t_LIM_ relationship, and used as the work rates for the “ON” bouts for the interval training sessions.


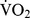
 in these tests was measured in the final minute of the 5.5 km·h^−1^ warm-up, thus establishing the baseline 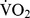. 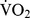
_peak_ was established by serial sampling of the expired gas (60 s collections) once the subject was considered to be close to the point of exercise intolerance (see Incremental-ramp test protocol above for further details). This 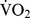
_peak_ was confirmed as 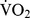
_max_ for each subject by establishing no difference in the 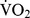
_peak_ attained with increases in constant-speed. Capillary blood samples were taken for lactate concentration ([L^−^]) analysis at rest, 30 s prior to the end of the 5.5 km·h^−1^ warm-up and immediately following the attainment of the limit of tolerance.

#### Fixed WR HIIT

A sub-group of 8 subjects completed a series of 3 HIIT sessions, one at WR_4_, WR_6_ and WR_8_, in a random order. Following the completion of the 5.5 km·h^−1^ warm-up, the work rate alternated between 4 min of the appropriate ON work rate (i.e. WR_4_, WR_6_, or WR_8_) and 4 min brisk walking at 5.5 km·h^−1^. This was repeated until the point of exercise intolerance, or until a maximum of 8 ON bouts were completed (Figure 1), allowing the total ON time, % of the target 16 min ON duration (i.e. 4 ON bouts of 4 min) to be calculated for each of the work rates performed.

#### Maximal HIIT

Given that effort is not maximal until the final bout in the fixed-WR HIIT protocol, a sub-group of 6 subjects completed a HIIT session in which the aim was to maximize effort in each of the 4 ON bouts, thus maximizing the amount of high-intensity work that can be completed with this format of training (i.e. analogous to SIT), with an anticipated duration of 4 min for each bout. The first ON bout was conducted at WR_4_ until the point of exercise intolerance was attained (at which point D′ is theorized to be fully ‘depleted’; [18], [31], [35]). The remaining 3 ON bouts were conducted at WR_8_ and continued until the point of exercise intolerance, with this theorized to result in a t_LIM_ of ∼4 min (based on evidence suggesting a D′ recovery of ∼50% with an intervening recovery of 4 min [35]; Figure 1). In each ON bout t_LIM_ was recorded and used to calculate the extent of D′ recovery in the preceding recovery period, and the amount of supra-CS work done for each bout.

During both HIIT protocols, “baseline” 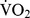 was measured in the final 60 s of the initial 5.5 km·h^−1^ warm-up, and in the final 60 s of each 4 min recovery between each ON bout. 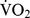
_peak_ was also measured in the final 60 s of each ON bout, with serial sampling conducted when the subject was considered to be close to their tolerable limit (see above) to ensure 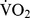
_peak_ was captured at the point of intolerance. Similarly, capillary blood samples were taken for [L^−^] analysis at rest, 30 s prior to the end of the 5.5 km·h^−1^ warm-up and 30 s prior to the onset of the next ON bout (“baseline”), immediately following the completion of each ON bout and immediately at the point of exercise intolerance. Subjects were informed during the HIIT protocols that if access to water was required this could be provided during the fixed 4 min recovery periods.

### Analysis

Normal data distribution was confirmed using Kolmogorov-Smirnov test. A one-way ANOVA for repeated measures, with post hoc analysis (bonferroni) where appropriate, was used to compare 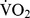
_peak_ and peak [L^−^] values obtained in all protocols and baseline 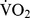 and [L^−^] values obtained during the maximal HIIT protocol. Similarly this test was used to compare the ON duration sustained during HIIT at WR_4_, WR_6_ and WR_8_, and the amount of supra-CS work performed during each interval during the maximal HIIT protocol. In addition, where appropriate, Cohen's d was used of provide a measure of the Effect size. The α was set at 0.050. Values are expressed as mean ± SD unless otherwise stated.

## Results

### Incremental-ramp test


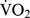
_peak_ (4.12±0.42 l·min^−1^; 57.6±4.3 ml·kg^−1^·min^−1^; Range 50.9–65.0 ml·kg^−1^·min^−1^) was attained at an average speed of 18.9±1.8 km·h^−1^ during the incremental-ramp test. Peak [L^−^] was 8.9±1.4 mM, and peak HR was 192±8 beats·min^−1^.

### Characterization of the S-t_LIM_ relationship

**Figure 2:**
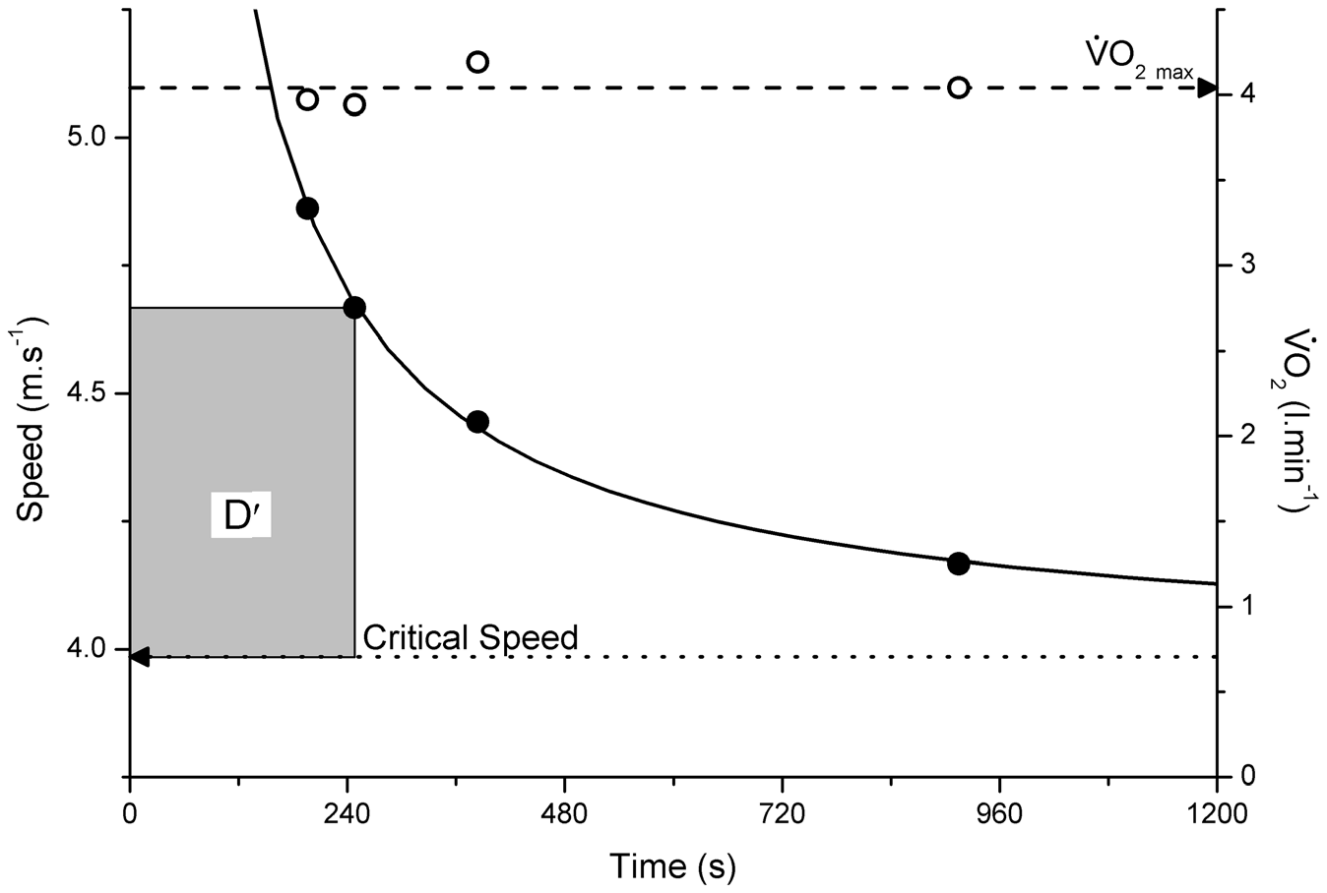
The relationship between speed and tolerable duration for 4 constant-speed tests (continued to exercise intolerance) in a representative subject ( •**).** A hyperbolic relationship has been fitted to these data (solid line) allowing estimation of critical speed and D′. Also plotted is the 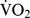 for each of these constant-speed tests (○), demonstrating 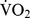
_max_ was attained in each test.

The individual values for 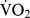
_peak_ were not influenced by treadmill speed (P>0.050), hence the mean of these values was taken as 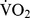
_max_ (4.13±0.39 l·min^−1^). Similarly, there was no difference in peak [L^−^] (P>0.050; mean 8.5±1.3 mM) or peak HR (P>0.050; mean 188±8 beats·min^−1^) with work rate at the point of exercise intolerance. Tolerable duration was well described by a hyperbolic function of the external treadmill speed, with the SE of the CS and D′ estimates of this relationship <0.06 m·s^−1^ (<2%; Range 0.3–1.8%) and <18 m (<10%; Range 1.6–7.9%), respectively, in all instances (Figure 2). CS and D′ averaged 3.853±0.429 m·s^−1^ (equivalent to 13.9±1.5 km·h^−1^) and 269.1±73.2 m, respectively. WR_4_, WR_6_ and WR_8_ interpolated from this S-t_LIM_ relationship were 4.974±0.527 m·s^−1^ (17.9±1.9 km·h^−1^), 4.600±0.475 m·s^−1^ (16.6±1.7 km·h^−1^) and 4.413±0.455 m·s^−1^ (15.9±1.6 km·h^−1^), respectively.

### Fixed WR HIIT

**Figure 3:**
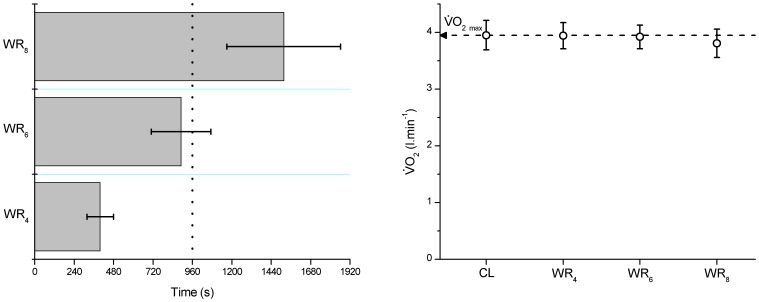
Left panel: The tolerable duration of HIIT at WR_4_, WR_6_ and WR_8_ (i.e. the work rate interpolated from the Speed-tolerable duration relationship to induced exercise intolerance in 4 min, 6 min and 8 min if performed at a constant speed), relative to the target HIIT duration of 960 s (i.e. 4×4 min; dotted vertical line). Right panel: The corresponding 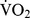 attained at exercise intolerance during HIIT at WR_4_, WR_6_ and WR_8_, relative to 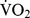
_max_ (dotted line), and that attained in the constant-speed (CL) tests. Note 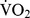
_max_ was attained in all protocols, although 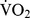 during HIIT at WR_8_ was slightly (insignificantly) lower due to some subjects being able to complete the maximum 8 bouts.

In the sub-group of 8 subjects who completed the 3 fixed work rate HIIT sessions at WR_4_, WR_6_ and WR_8_ the tolerable duration of the HIIT sessions were 399±81 s (95% CI; 331–467 s), 892±181 s (95% CI; 741–1044 s), and 1517±346 s (95% CI; 1228–1807 s), respectively with total ON durations all significantly different from each other (P<0.050) (Figure 3A). This was equivalent to 41.6±8.4% (95% CI; 34.5–48.6%), 93±18.9% (95% CI; 77.2–108.8%) and 158.1±36.1% (95% CI; 127.9–188.2%) of the target 960 s (i.e. 4×4 min) ON duration. There was, however, no difference in the 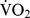 attained at the limit of tolerance of WR_4_, WR_6_ or WR_8_ protocols, with this 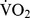 not different from 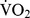
_max_ in this cohort of 8 subject (P>0.050), thus confirming 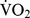
_max_ was attained in all protocols. However, there was a tendency for the 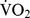 attained during WR_8_ to be lower than 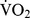
_max_ (Cohen's d = 0.55) due to some subjects being able to complete the maximum 8 ON bouts, hence these subjects did not attain the point of exercise intolerance before the protocol was terminated (Figure 3B; Table 1). Similarly, there was no difference in peak HR at the point of exercise intolerance in all protocols (P>0.050; Table 1). In addition, peak [L^−^] was not significantly different from that attained during the constant-speed tests used to characterize the S-t_LIM_ relationship at the point of exercise intolerance in WR_6_ and WR_8_ (P>0.050); however, peak [L^−^] was significantly higher in WR_4_ at the point of exercise intolerance than that attained in all other protocols (P<0.050; Table 1). Thus WR_6_ provides the appropriate work rate to normalize the intensity of HIIT to the very-heavy intensity domain, with this speed equivalent to 88±3% (Range 83–93%) of the speed attained at 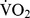
_max_ in the incremental-ramp test.

**Table 1:** 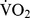
_max_, HR and [L^−^] attained at the limit of tolerance during Control, and fixed WR HIIT protocols performed at WR_4_, WR_6_ and WR_8_.

	Control	WR_4_	WR_6_	WR_8_
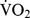 _max_ (l·min^−1^)	3.95±0.26	3.94±0.23	3.92±0.21	3.81±0.25
HR (beats·min^−1^)	188±43	190±3	189±6	188±4
[L^−^] (mM)	8.4±1.1	9.7±1.1*	8.5±1.5	8.0±1.0

Values are means ± SD. 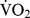
_max_ (maximal rate of pulmonary oxygen uptake); HR (heart rate) and [L^−^] (Lactate concentration) measured at the limit of tolerance during the constant-speed tests used to characterize the Speed-tolerable duration (S-t_LIM_) relationship (Control) and during HIIT performed at WR_4_, WR_6_ and WR_8_ (work rates predicted to induce exhaustion at 4, 6 and 8 min respectively).

* Significantly higher [L^−^] than that attained in Control, WR_6_ and WR_8_ protocols.

### Maximal HIIT

**Figure 4:**
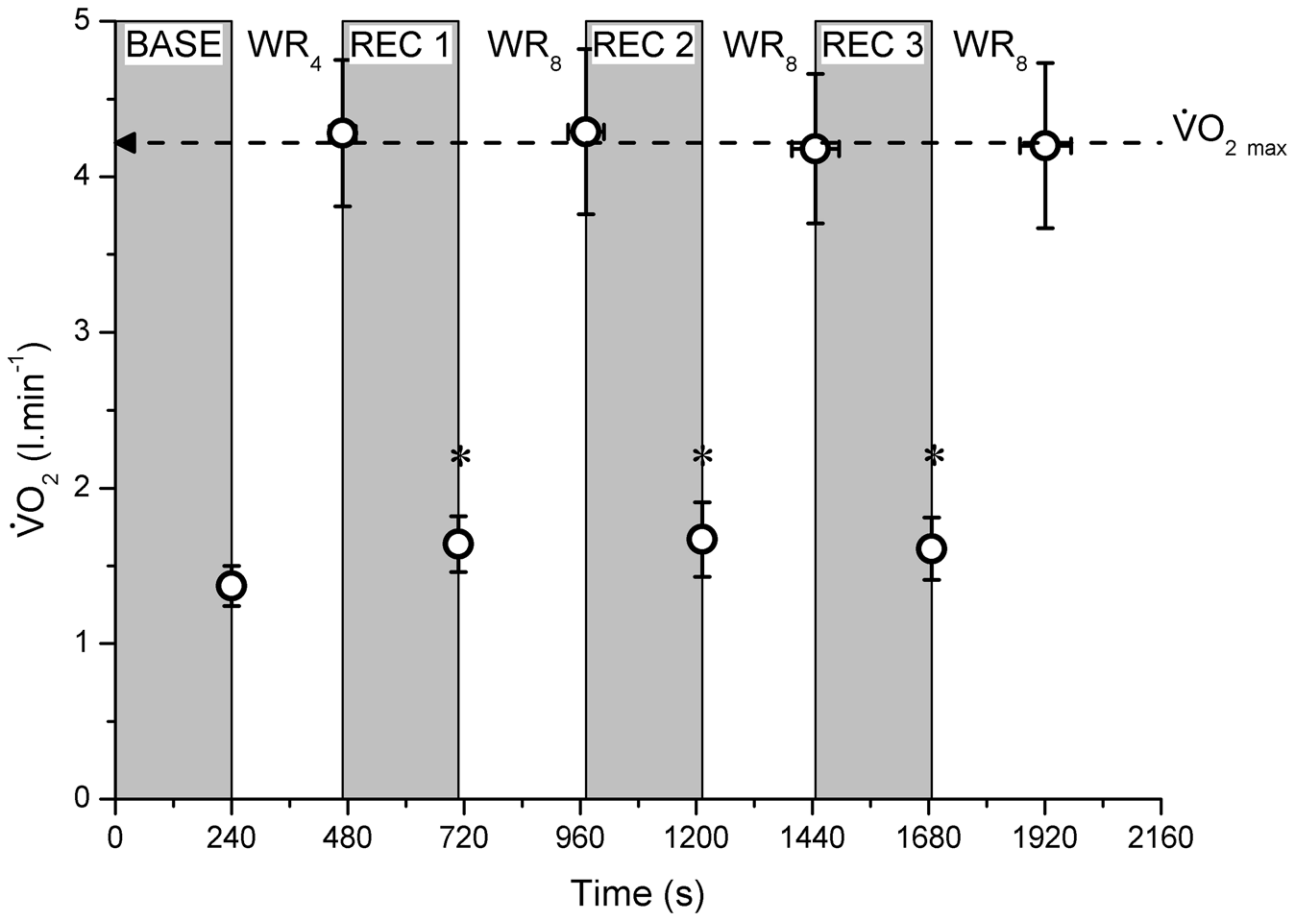
The 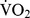 response during the maximal HIIT protocol. Although there was some (insignificant) variability in ON duration at WR_4_ (bout 1) and WR_8_ (bouts 2, 3 and 4) (horizontal error bars), note the constancy of the 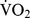 attained, with this indistinguishable from 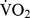
_max_. Similarly, although 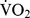 did not recover to baseline (BASE) following 4 min recovery (REC; P<0.050, ★), there was no difference in the 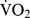 attained in REC following WR_4_ (bout 1) or WR_8_ (bouts 2 and 3).

In the sub-group of 6 subjects who completed this protocol, there was no significant difference in tolerable duration for each of the 4 ON bouts (ON Bout 1: 229±27 s; Bout 2: 262±37 s; Bout 3: 235±49 s; Bout 4: 235±53 s; P>0.050); with 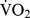
_max_ attained in each of the 4 ON bouts (Table 2; Figure 4). Although there was a statistical difference in the 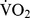 attained at the point of exercise intolerance between ON bouts 2 and 3 (P = 0.047), neither of these was different from 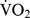
_max_ determined during the constant-speed tests (P>0.050), and there was less than a 0.20 l·min^−1^ difference between all measurements of 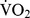 at the point of exercise intolerance in all subjects and 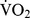
_max_. Hence, 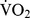 remained well within the expected test-retest variability of 10% [41]. Similarly, there was no difference in HR at the point of exercise intolerance between each of the ON bouts, or that attained during the constant-speed tests (P>0.050; Table 2). In addition, although there was a significant difference in peak [L^−^] between ON bouts 1 and 2 (P<0.050), there was no difference in peak [L^−^] between all other bouts, and that attained in the constant-speed tests (P>0.050; Table 2).

**Table 2:** 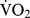
_max_, HR and [L^−^] attained at the limit of tolerance during the control and maximal HIIT protocols.

	Control	ON Bout 1	ON Bout 2	ON Bout 3	ON Bout 4
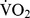 _max_ (l·min^−1^)	4.22±0.51	4.28±0.47	4.29±0.53	4.18±0.48	4.20±0.53
HR (beats·min^−1^)	190±3	188±6	185±9	189±5	188±6
peak [L^−^] (mM)	8.2±1.1	7.2±1.5*	9.7±1.6	9.3±1.2	9.1±1.4

Values are means ± SD. 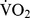
_max_ (maximal rate of pulmonary oxygen uptake); HR (heart rate) and [L^−^] (Lactate concentration) measured at the limit of tolerance during the constant-speed tests used to characterize the Speed-tolerable duration (S-t_LIM_) relationship (Control) and ON bouts 1, 2, 3 and 4 of the maximal HIIT protocol.

* Significantly lower [L^−^] than that achieved in Bout 2.


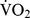
 prior to each ON bout was elevated compared to the pre-exercise baseline value (P<0.050), however this was not significantly different between recovery (REC) bouts (P>0.050; Table 3, Figure 4). Similarly HR was elevated compared to the pre-exercise baseline value (P>0.050); however, there was no significant difference between the HR attained during the recovery bouts (Table 3). In addition, [L^−^] was significantly elevated prior to the pre-exercise baseline value in all recovery bouts (P>0.050); however, there was no significant difference in [L^−^] attained between each intervening recovery bout (P>0.050; Table 3).

**Table 3:** 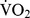
_max_, HR and [L^−^] attained at the pre-exercise baseline, and in the 4 min recovery bouts during the maximal HIIT protocol.

	Baseline	REC 1	REC 2	REC 3
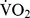 _max_ (l·min^−1^)	1.37±0.13	1.64±0.18*	1.67±0.24*	1.61±0.20*
HR (beats·min^−1^)	108±6	132±7*	133±7*	137±8*
peak [L^−^] (mM)	0.9±0.2	9.2±1.4*	8.8±1.3*	8.6±1.9*

Values are means ± SD. 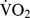
_max_, maximal rate of pulmonary oxygen uptake; HR, heart rate; [L^−^], Lactate concentration and; REC, recovery bout.

* Significantly higher than the pre-exercise baseline.

**Figure 5:**
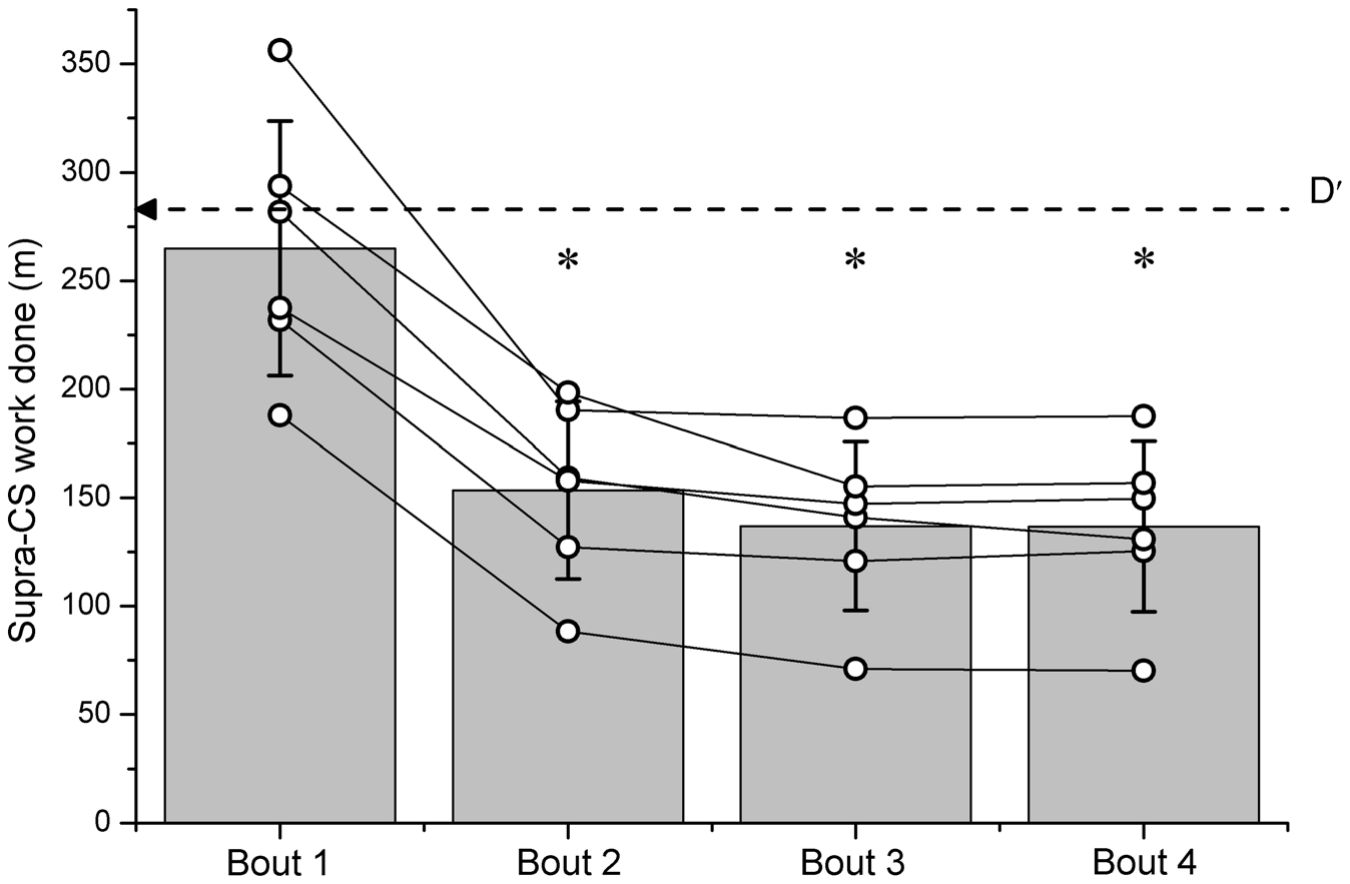
The quantity (with units of meters, m) of supra-CS work (i.e. D′) performed during the maximal HIIT protocol. Bars represent the group mean (± SD), with ○ representing the individual data. In bout 1, the amount of supra-CS work performed is indistinguishable from D′ determined from the Speed-tolerable duration relationship. However, in bouts 2, 3 and 4 significantly less supra-CS work is performed (∼50%) with this significantly less than D′ (P<0.050, ★).

These results are consistent with the finding that there was no difference in supra-CS work in Bout 1 compared with D′ (264.9±58.7 m vs. 283.7±79.5 m; P>0.050), but that there was significantly less work accomplished in ON bouts 2, 3 and 4 (P<0.050), with the amount of supra-CS work not different between these bouts (Bout 2: 153.5±40. 9 m; Bout 3: 136.9±38.9 m; Bout 4: 136.7±39.3 m; Figure 5; P>0.050) suggesting a constant *rate* of D′ ‘recovery’ – thus fixed *quantity* of D′ recovered in 4 min – between bouts. D′ recovery averaged 54.7±7.8%, 48.9±10.2% and 48.9±11.2% for bouts 2, 3 and 4 respectively, with this recovery not significantly different between bouts (P<0.050).

## Discussion

This is the first study to apply the S-t_LIM_ relationship to identify the appropriate work rate for HIIT to normalize the relative intensity between subjects to the very-heavy intensity domain, identifying that WR_6_ for a 4×4 min HIIT session provides the appropriate balance between D′ depletion during the ON bouts, and repletion in the intervening 4 min recovery period that allowed for the completion of the required ∼4 (3.7±0.7; i.e. 93%) ON bouts. Hence, this protocol allows for the appropriate consideration of the role of exercise intensity in determining training adaptations, normalizing this between individuals. Furthermore, this study establishes a protocol that, with knowledge of the extent of D′ recovery between bouts, maximizes the amount of high-intensity work that can be completed in a 4×4 min HIIT protocol, precisely normalizing the intensity of both the overall session, and each ON bout (i.e. each ON bout resulted in the attainment of 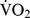
_max_). Hence, this protocol provides a means of differentiating the relative importance of the work rate profile (c.f. SIT) and exercise intensity to promote physiological adaptations.

### Exercise intensity

While a specific work rate can be of a high absolute intensity (e.g. 100% 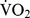
_max_) when performed as a continuous bout, this same specific work rate can be undertaken during HIIT in a manner which means the overall intensity of the training session can be either moderate, (metabolic rate<Lactate threshold (LT), no sustained metabolic acidosis), heavy (metabolic rate>LT<CS/CP, sustained metabolic acidosis which eventually attains a steady-state) or very-heavy/severe (progressive increase in 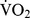, resulting in the attainment of 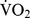
_max_ if continued to t_LIM_, progressive metabolic acidosis which continues throughout the exercise until t_LIM_) [19], [42]. While the specific work rate performed in relation to the overall intensity of training is not a consideration in short-duration SIT, as the ∼30 s sprints are an all-out effort (e.g. [20], [21], [22], [23], [24]) with this long enough to result in the attainment (or very near attainment) of HR_max_ and 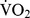
_max_ in each sprint (i.e. very-heavy/severe intensity), the specific work rate used during HIIT is an essential consideration with respect to exercise intensity (and normalizing this between participants) when the duration of the ON exercise bout is extended.

Exercise bouts of 4 min are frequently used in HIIT both for health and performance benefits (e.g. [9], [10], [12], [29], [30]) in the format of a 4×4 min training session, with an intervening recovery of 3–4 min. Typically work rate is determined from % HR_max_ or % 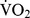
_max_; however, this fails to account for the variability of the derived work rate with respect to the parameters of the high-intensity relationship (i.e. CS and D′) between individuals (e.g. [19], [39]). This is highlighted by the result in this study that WR_6_ exists at 87% of the speed attained at 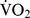
_max_ in the incremental-ramp test, but with a range of 83–93%. In addition, any specific prescribed % 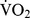 or % HR during HIIT is only attained fleetingly as a steady-state is never achieved, with these variables continuing to increase towards their respective maxima throughout each bout [19]. However, by accounting for the S-t_LIM_ relationship during treadmill running to normalize exercise intensity we were able to demonstrate that WR_6_ (i.e. the work rate derived from the S-t_LIM_ relationship that leads to the limit of tolerance in 6 min) was optimal, providing the required balance between D′ “depletion” during ON bouts and “repletion” during the intervening recovery that allowed for the completion of the required ∼4 ON bouts. As this resulted in the attainment of 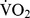
_max_ and peak lactate in the final bout this, by definition [19], [42], puts the overall intensity of training for all subjects within the very-heavy intensity domain.

### Maximal HIIT

While WR_6_ defines a work rate for HIIT that normalizes intensity to the very-heavy intensity domain for 4×4 min HIIT, with this resulting in a high 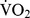 and HR (with these variables continuing to increase towards their respective maxima throughout each bout until the limit of tolerance is attained), maximal effort is not required until the final bout (c.f SIT; [43]). Therefore, while WR_6_ normalizes exercise intensity it does not maximize the amount of supra-CS work that can be completed during 4×4 min HIIT. For high-intensity, supra-CS exercise of ∼2–30 min tolerable duration is dependent on the rate of D′ depletion, with this rate of depletion increasing proportionally with work rate. Therefore, interpolating WR_4_ from the S-t_LIM_ relationship maximizes supra-CS work on Bout 1 of 4×4 min HIIT (Bout 1 t_LIM_: 229±27 s; Range 117–248 s), with this leading to the attainment of HR_max_ and 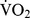
_max_. As CS is unchanged following fatiguing exercise, subsequent exercise tolerance is dependent exclusively on the extent of D′ recovery, with this demonstrated to be ∼50% in 4 min recovery [35]. Hence, in Bout 2 WR_8_ should be sustainable for ∼4 min, thus providing the necessary work rate to maximize supra-CS work in 4 min. In this study as D′ recovery averaged ∼50%, and confirms the assumption that the extent of D′ recovery does not differ between repeated bouts [44], WR_8_ is then the appropriate work rate for bouts 2, 3 and 4 to maximize the amount of supra-CS work that can be accumulated in 4 min (t_LIM_: Bout 2: 262±37 s; Bout 3: 235±49 s; Bout 4: 235±53 s), and provides a protocol to maximize the amount of supra-CS work that can be accumulated in 4×4 min HIIT, resulting in the attainment of HR_max_ and 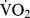
_max_ in each bout. This makes this 4×4 min HIIT analogous to SIT, allowing the relative contribution of the work rate profile, when matched for exercise intensity, to be investigated. These data also confirm the assumption that during this 4×4 min HIIT protocol performance is determined by the S-t_LIM_ relationship, with the profile of D′ depletion and recovery alone “shaping” supra-CS exercise tolerance [44].

### Consideration of the work rate profile of HIIT and practical applications

By correctly defining and normalizing the intensity of HIIT between participants to maximize the amount of supra-CS work that can be accumulated in a 4×4 min HIIT protocol, thus ensuring the same metabolic stress throughout training, this allows appropriate comparison of different interval training strategies (e.g. short vs. long duration ON bouts). Hence, the relative contributions of both exercise intensity and the intermittent/interval work rate profile to any training induced physiological adaptations can be appropriately deconvoluted, with this having important implications when investigating the mechanistic basis for training adaptations.

While exercise intensity is an essential consideration with regards to training adaptations [16], [30], [45], [46], [47] there is evidence emerging that the actual work rate profile is also important [12], [28]. Even when appropriately matching for exercise intensity and total work the physiological changes during HIIT (in terms of, for example, the dynamics and proportional contribution of the different energy systems to the energy demand, and blood flow dynamics) will be significantly different with short, compared with long ON bouts. That is, when the overall intensity of the exercise session is controlled, but the duration of the ON bout is extended, there is a proportionally greater aerobic contribution to the overall energy requirement when matched for energy expenditure. Hence with longer ON bouts (i.e. ∼4 min), given the response dynamics of 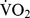, HR and cardiac output, there will be a greater time accumulated at a relatively high proportion of these respective maxima, compared with short (i.e. 30 s) ON bouts. Although this requires further systematic investigation, this is likely to have significant consequences with regards to the specific physiological adaptations seen (e.g. [28]) following a training program.

For example, it has been suggested that there may be an intensity threshold over which exercise has to be performed to promote cardiovascular benefits [13], [14], although this suggestion is not universal (e.g. [17]). Hence, it is likely that generating a high relative HR and cardiac output (with respect to their maxima) is important for inducing intrinsic cardiac benefits and promoting improvements in vascular function [9], [13], [45]. Thus, as there is a greater accumulation of time under these “conditions” i.e. high HR and cardiac output in long vs. short bouts for the same overall training session intensity and total training session time commitment (i.e. ∼30 min per session), the relevance of the work rate profile is likely an essential consideration with regards to developing optimal training strategies to maximize training adaptations.

In addition, the physiological differences between different HIIT protocols, even when matched for exercise intensity may be of particular importance when considering adaptations relating to metabolic and cardiovascular risk factors such as insulin sensitivity and aerobic capacity. For example, increased mitochondrial energy flux is associated with greater improvements in insulin sensitivity [48]. In addition, while PCG-1α (a critical regulator of mitochondrial biogenesis; [49]) has been demonstrated to be activated following both short [50], [51] and long [11] HIIT, given the bioenergetic differences between the different interval training strategies it is unclear which work rate profile will have the greatest impact on, for example, mitochondrial capacity, insulin sensitivity and aerobic capacity when exercise intensity is controlled. Therefore, while the work rate profile likely contributes to training adaptations, the *specific* work rate profile which maximizes *specific* adaptations to subsequently improve physiological function (and the interaction of this with the exercise intensity) has yet to be resolved. However, the results from this study enable the correct work rates to be identified, thus allowing intensity to be removed as a confounding variable in order to investigate the relative importance of the work rate profile in training strategies.

While it has been postulated that there is a dose-response relationship between exercise intensity (quantified in terms of 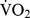
_max_) and training adaptations [14], the full nature of this dose-response has yet to be established. Therefore, whether it is necessary to provide an all-out effort to maximize any physiological adaptations from training has yet to be resolved. Hence, it is possible that, similar to the proposal that there is a minimum intensity for some specific training induced adaptations [13], [14], there may also be an upper limit/optimal exercise intensity above which the magnitude of any training induced adaptations is diminished. However, calculating appropriate work rates based on the S-t_LIM_ relationship and the extent of D′ recovery, provides a method of normalizing, and then titrating the overall training intensity for longer duration ON bouts to identify the existence of any intensity (comparing this with appropriate proposed “practical” short duration HIIT strategies; e.g. [52], [53]), and thus effort related threshold with regards to training adaptations. This however, requires systematic investigation.

Of course underpinning all considerations with regards to training strategies and physiological adaptations must be the potential to translate effective lab-based training strategies in to the home environment. There is evidence that interval training in general may be a more enjoyable training strategy than continuous moderate-intensity interventions [37], with this possibly related to the challenge of undertaking the more challenging aspect of the exercise intervals, rather than the monotony of continuous moderate-intensity exercise. Hence, research into optimizing HIIT has the potential to have a significant impact with regards to establishing a range of effective training strategies as alternatives to traditional continuous moderate-intensity exercise for the improvement of exercise tolerance/performance and risk factors for chronic illness, allowing individuals to adhere to training strategies which fit with their individual training preferences and lifestyle [54].

### Limitations

While this study has identified that WR_6_ normalizes the intensity of HIIT to the very heavy-intensity domain, it must be acknowledged that the subjects in this study were from a relatively homogenous group; therefore, whether these findings can be extended to other populations remains unclear. In addition, although these results highlight the importance of the S-t_LIM_ relationship to normalize the intensity of both continuous and HIIT one of the primary limitations when considering the translational application of these findings is the number of tests required to characterize this relationship and identify the correct work rates. Therefore, developing a strategy that allows quick and accurate identification of the appropriate work rates from the S-t_LIM_ relationship for use in HIIT remains an important goal.

### Conclusion

In conclusion, WR_6_ derived from the S-t_LIM_ relationship provides the appropriate work rate to normalize the intensity of 4×4 min HIIT to the very heavy-intensity domain. In addition, as there is no difference in the extent of D′ recovery between fatigue bouts, this study establishes an approach in which supra-CS work can be maximized and exercise intensity can be normalized precisely for each subject during 4×4 min HIIT. This strategy therefore allows the relative contributions of exercise intensity and the work rate profile to any training induced adaptations to be appropriately quantified. This has important implications for establishing HIIT strategies to maximize improvements in physiological functioning.
